# What is Your Diagnosis?

**DOI:** 10.5812/iranjradiol.7695

**Published:** 2012-06-30

**Authors:** Abbas Khodayari Namin, Sanam Mirbeigi, Fereshteh Ensani

**Affiliations:** 1 Department of Oral and Maxillofacial Surgery, School of Dentistry, Shahid Beheshti University of Medical Sciences, Tehran, Iran; 2 Department of Oral and Maxillofacial Radiology, School of Dentistry, Kerman University of Medical Sciences, Kerman, Iran; 3 Department of Pathology, Medical School, Tehran University of Medical Sciences, Tehran, Iran

A 9-year-old white female with a round, immobile, tender and firm swelling measuring about 1.5 cm on the right side of the mandibular body, was referred for extraction of the related teeth (Figure 1). There was no fistulae, pus and color change of the covering skin. Paresthesia and anesthesia were not found either. There were nontender lymph nodes at the left side of the mandible, right submaxillary and anterior to the sternocleidomastoid muscle. The laboratory tests were normal except for a high ESR.

**Figure 1:**
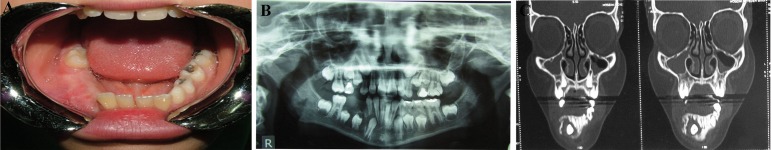
A, On intraoral examination, expansion of the buccal and lingual cortex of the right mandibular body with more buccal extension of the lesion was observed. The lesion measured approximately 4 cm anteroposteriorly and 2 cm buccolingually; B, Panoramic radiograph discloses a permeative, poorly demarcated, destructive lesion in the right mandibular body from the right permanent first molar to the right lateral incisor;C & D, Reconstructed CT scan of the head and neck shows a lytic destructive lesion with cortical expansion and destruction from the right permanent first molar to the right lateral incisor.

## Diagnosis: Ewing’s Sarcoma Mimicking Dentoalveolar Abscess

**Figure 2:**
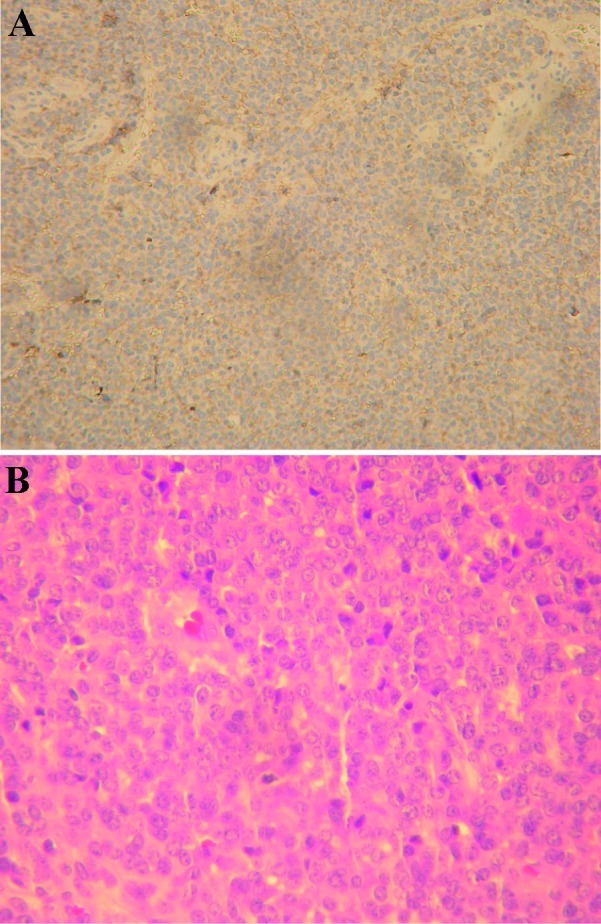
A and B: Histopathological studies included demonstration of glycogen by PAS and immunohistochemical staining

Immunohistochemistry indicated that tumor cells were positive for Vimentin and CD99 (Mic-2). They were negative for Desmin, Myogenin, Myo-D, CD20, CD3 and CD79a. Proliferative activity (Ki67) was significant (40% nuclear staining) (Figure 2). The diagnosis was malignant small round cell tumor with bone and soft tissue involvement consistent with Ewing’s sarcoma. Ewing’s sarcoma is the second most common primary malignant tumor of the bone found in children (4-6%) after osteosarcoma (1-5). Although Ewing’s sarcoma can occur in any part of the skeleton, it shows a particular predisposition for the long bones of the extremities and pelvis, which respectively account for 58% and 20% of all documented cases (6-9). Swelling of the involved area, pain and high ESR are the most common presenting signs and symptoms for mandibular Ewing’s sarcoma similar to this case (10).

Generally, on plain radiography, Ewing’s sarcoma presents as a radiolucency that is poorly demarcated and never corticated. Its advancing edge destroys the bone in an uneven fashion, resulting in a ragged border (11). The lesions are usually solitary with adjacent radiographically visible soft tissue masses (11). They may be round or ovoid but generally have no typical shape. Ewing’s sarcoma is a destructive process with little induction of bone formation. Because it commences on the internal aspect of the bone and involves the endosteal and periosteal surfaces later in its course, it is usually entirely radiolucent. Ewing’s sarcoma may stimulate the periosteum to produce new bone. However, “onion skin” periosteal reaction, which is a common finding in long bones, occurs rarely in the mandible (11). In the mandible, it usually takes the forms of “Codman’s triangle “or “sunray” or “hair-on-end spiculation” (8). Laminar periosteal new bone formation has been reported to occur, but it is not a common finding. Adjacent normal structures such as the inferior mandibular canal, the inferior border of the mandible and the alveolar cortical plates may be affected (6-8). If the lesion abuts teeth or tooth follicles, the cortices of these structures are destroyed. This tumor does not characteristically cause root resorption, although it does destroy the supporting bone of adjacent teeth. Conventional radiographs are not specific; therefore, a differential diagnosis should also include pyogenic osteomyelitis, osteosarcoma, eosinophilic granuloma, chondrosarcoma, fibrosarcoma and metastatic lesions (11). Inflammatory or infectious lesions such as osteomyelitis of the jaw may show some of the radiographic features of Ewing’s sarcoma. With similar clinical symptoms the first differential diagnosis could be these type of lesions. Although both are radiolucent, osteomyelitis is likely to have demonstrable sequesters present within the confines of the lesion on CT scan, whereas Ewing’s sarcoma does not. Inflammatory lesions contain some sign of reactive bone formation resulting in some sclerosis internally or at the periphery and they differ in the associated periosteal bone formation. It is probable that inflammatory conditions superpose on the tumor, but complete investigation of the lesion is necessary and the unusual response of the lesion to antibiotics could suggest pathogenesis other than simple infection.

CT scan with higher resolution than conventional radiography demonstrating the periosteal new formation in the form of sunray appearance. MRI is today the method of choice for staging Ewing’s sarcoma (11). It is also a good imaging technique for monitoring the effect of therapy since it can help identify the presence of a recurrent or residual tumor (11). In this case, MRI was requested 5 months after surgery for better monitoring of the patient. No specific radiographic features have been reported in the literature to distinguish Ewing’s sarcoma in children as osteosarcoma or other malignancies (8). Definitive diagnosis relies on histology.

## References

[1] Bessede JP, Monsaint B, Huth J, de Lumley L, Sauvage JP (1993). Ewing’s sarcoma of the mandible: a case report. Br J Oral Maxillofac Surg.

[2] Ewing J (1921). Diffuse endothelioma of bone. proc.

[3] Girish H, Umadevi H, Priya N, Sharada P (2006). Ewing’s sarcoma of the mandible.

[4] Infante-Cossio P, Gutierrez-Perez JL, Garcia-Perla A, Noguer-Mediavilla M, Gavilan-Carrasco F (2005). Primary Ewing’s sarcoma of the maxilla and zygoma: report of a case. J Oral Maxillofac Surg.

[5] Vaccani JP, Forte V, de Jong AL, Taylor G (1999). Ewing’s sarcoma of the head and neck in children. Int J Pediatr Otorhinolaryngol.

[6] Berk R, Heller A, Heller D, Schwartz S, Klein EA (1995). Ewing’s sarcoma of the mandible: a case report. Oral Surg Oral Med Oral Pathol Oral Radiol Endod.

[7] Wang CL, Yacobi R, Pharoah M, Thorner P (1991). Ewing’s sarcoma: metastatic tumor to the jaw. Oral Surg Oral Med Oral Pathol.

[8] Wood RE, Nortje CJ, Hesseling P, Grotepass F (1990). Ewing’s tumor of the jaw. Oral Surg Oral Med Oral Pathol.

[9] Hardy P, Gibbs AR (1984). Ewing’s sarcoma of mandible. Br J Oral Maxillofac Surg.

[10] Dehner LP (1993). Primitive neuroectodermal tumor and Ewing’s sarcoma. Am J Surg Pathol.

[11] Gorospe L, Fernandez-Gil MA, Garcia-Raya P, Royo A, Lopez-Barea F, Garcia-Miguel P (2001). Ewing’s sarcoma of the mandible: radiologic features with emphasis on magnetic resonance appearance. Oral Surg Oral Med Oral Pathol Oral Radiol Endod.

